# Respiratory Infections in Children During a Covid-19 Pandemic Winter

**DOI:** 10.3389/fped.2021.740785

**Published:** 2021-10-18

**Authors:** Susanne C. Diesner-Treiber, Peter Voitl, Julian J. M. Voitl, Klara Langer, Ulrike Kuzio, Angela Riepl, Pia Patel, Alexandra Mühl-Riegler, Bernhard Mühl

**Affiliations:** ^1^First Vienna Pediatric Medical Center, Vienna, Austria; ^2^Medical University of Vienna, Vienna, Austria; ^3^Sigmund Freud University Vienna, Vienna, Austria; ^4^Vienna Medical Innovation Center, Vienna, Austria

**Keywords:** SARS-CoV-2, Covid-19 mitigating measures, respiratory pathogens, multiplex PCR, pediatrics

## Abstract

**Background:** The Covid-19 pandemic compelled the implementation of measures to curb the SARS CoV-2 spread, such as social distancing, wearing FFP2 masks, and frequent hand hygiene. One anticipated ramification of these measures was the containment of other pathogens. This prospective, longitudinal study aimed to investigate the spread of 22 common seasonal non-SARS-CoV-2 pathogens, such as RSV and influenza, among children with an acute respiratory infection during a pandemic.

**Methods:** Three hundred ninety children (0-24 months) admitted to Vienna's largest pediatric center with acute respiratory infection (November 2020-April 2021) were included in this study. The researchers tested nasal swabs for 22 respiratory pathogens by Multiplex PCR, documented clinical features and treatment, and evaluated data for a potential connection with the lockdown measures then in force.

**Results:** The 448 smears revealed the most common pathogens to be rhino-/enterovirus (41.4%), adenovirus (2.2%), and coronavirus NL63 (13.6%). While the first two were active throughout the entire season, coronaviruses peaked in the first trimester of 2021 in conjunction with the lift of the lockdown period (OR 4.371, 95%CI 2.34-8.136, *P* < 0.001). RSV, metapneumovirus, and influenza were absent.

**Conclusion:** This prospective, longitudinal study shows that Covid-19 measures suppressed the seasonal activity of influenza, RSV, and metapneumovirus among very young children, but not of rhino-/enterovirus and adenovirus. The 0-24 month-olds are considered the lowest risk group and were only indirectly affected by the public health measures. Lockdowns were negatively associated with coronaviruses infections.

## Introduction

Respiratory infections are among the leading causes of children's admission to pediatric practices. Pathogens typically circulating in the winter can trigger similar symptoms in children (1) and, in severe cases, lead to hospitalization. The most commonly identified respiratory pathogens are rhinovirus/enterovirus, respiratory syncytial virus (RSV), adenovirus, influenza virus, parainfluenza viruses, and coronaviruses (2). While adenovirus occurs more often in children aged 3-6 years, RSV is more likely to affect infants and toddlers <3 years of age (3). Overall, infections with these pathogens thrive in winter, although seasonality may vary. While rhinovirus, adenovirus, and metapneumovirus are present throughout the year, influenza, RSV, and human coronaviruses (229E, HKU1, NL63, and OC43) have significantly higher communicability in winter (3, 4). In Austria, the RSV season ranges from October to the beginning of April, peaking around the 8th calendar week, while the influenza season usually falls in the first calendar weeks of the year, showing a distinct peak in the 5th calendar week of 2020 (5, 6).

Since the beginning of 2020, the world has been dealing with a new pandemic caused by SARS-CoV-2. At the time of our study, children typically had a milder clinical course, and their role in the transmission of the virus remained controversial (7). With all the numerous regulations worldwide, such as lockdowns, social distancing, hand hygiene, wearing mouth and nose protection (8, 9), the pandemic had a profound impact on public awareness of viral infections, and retrospective analyses on Covid-19 preventive measures showed a significant influence on the occurrence of co-infections. In the general population, Austria registered fewer rhinovirus, metapneumovirus, influenza, and RSV cases during the first lockdown in spring 2020 (10). In Italy, other infectious diseases such as varicella, pertussis, measles, and rubella were also less common (11).

Based on these observations, this longitudinal, prospective study aimed to investigate how the incidence of 22 seasonal non-Covid-19 respiratory pathogens developed in a representative Austrian pediatric cohort during a pandemic in a primary pediatric health care setting. Virological diagnostics specifically did not include SARS-CoV-2, as the health authorities are gathering data on the temporal course of its spread and connection with the current measures.

## Materials and Methods

### Study Design

This prospective, longitudinal study was conducted at the “First Vienna Pediatric Medical Center” between November 2020 and April 2021, the first whole winter semester with the Covid-19 pandemic in Austria. The study center is Austria's largest pediatric outpatient practice, with more than 57.000 patient contacts per year, or over 16% of all outpatient contacts in Vienna (2015) (12, 13). Its broad range of medical specialties guarantees healthcare to patients coming from the whole region.

### Study Population and Documentation of Patients' Characteristics

All families of children (0-24 months) meeting the inclusion criteria were asked to participate in this study. Eligible children presented with acute respiratory infection with at least one of the following symptoms: rhinitis, nasal congestion, cough, sore throat or fever of unknown origin (e.g., without gastroenteritis or urinary tract infection) with a maximum duration of one week. Preterm infants, children with severe congenital or chronic diseases (e.g., severe congenital heart malformations, severe pulmonary or neuromuscular diseases, severe immunodeficiency, and chromosomal aberration) were excluded.

The study was approved by the Ethics Committee of the Medical University of Vienna (EK-Nr. 1864/2020). Parents received extensive information on the objective and procedures of this study and provided written consent.

The case report form included the family socio-economic status (education of parents, housing situation, marital status, number of siblings), patient's general clinical data (weight, height, calculated BMI, chronic diseases), clinical features of the acute respiratory infection (rhinitis, nasal congestion, fever, fever duration, cough, pharyngitis), and treatment recommendations (decongestant nose spray, hypertonic saline nose spray, analgesics, antibiotics, inhalation therapy [salbutamol as short- acting beta mimetics (SABA), inhaled corticosteroids (ICS)], inhalation with sodium chloride), systemic corticosteroids, as well as indications for hospitalization if necessary.

We repeated nasal swabs in case of recurrent acute infection with healthy periods in-between. Socio-demographic and patient-specific data, however, were only collected at the first visit.

### Study Procedure

Nasal swab samples were collected by a trained study team from both nostrils and put into 0.9% sodium chloride until further analysis. The samples were screened using the Multiplex PCR respiratory panel (Biofire FilmArray Respiratory panel 2 plus kit) to detect adenovirus, coronaviruses (229E, HKU1, OC43, NL63), metapneumovirus, rhinovirus/enterovirus, influenza A (H1, H1-2009, H3), influenza B, parainfluenza 1-4, RSV, MERS-CoV, Chlamydia pneumoniae, Mycoplasma pneumoniae, Bordetella pertussis, Bordetella parapertussis, with high sensitivity and specificity for nasopharyngeal swabs (14). As recommended by the Ethics Committee, we performed the less invasive method of nasal swabs to reduce the discomfort of the procedure.

Samples were not tested for SARS-CoV-2 in this trial. However, in case of anamnestic or clinical signs of a possible SARS-CoV-2 infection, patients were referred to the official SARS-CoV-2 testing units.

### Covid-19 Regulations

Information on the Austrian Covid-19 regulations was constantly monitored on the homepage of the Federal Ministry of Social Affairs, Health, Care and Consumer Protection (9).

The numerous regulations to limit the circulation of the pathogen implicate physical distancing, temporary lockdowns, working from home, distance learning, kindergarten emergency assistance, the closure of shops not providing essential services, ban on travel, limitations to the foodservice industry, and curfews (8). Furthermore, it was mandatory to wear mouth and nose protection in certain public areas. In addition, the population was encouraged to disinfect their hands regularly (9).

In detail and for statistical analysis, lockdowns were either total lockdowns (calendar weeks 47-50, 53-6, and 13-16) which entailed a 24 h curfew and the cessation of cultural and leisure activities and events, the closure of hotels and hospitality facilities, or partial lockdowns (weeks 45-47, weeks 50-52) with a night-time curfew and the cessation of the above mentioned non-essential services but allowing retailers and service providers to remain open. During partial lockdowns, senior year students attended remote learning classes, which was then extended to all grades during total lockdowns. Attendance was not mandatory for kindergartens; however, childcare options were available for parents who could not look after their children at home. In-between lockdowns (calendar weeks 7-12), schools, shops, and museums were allowed to reopen, whereas the hospitality and food sectors remained closed.

From September 2020, mouth-nose protections and from January 2021 particle filtering half-masks (FFP2) were mandatory in public indoor places, such as shops, public transportation, indoor markets, gastronomy, and administrative authorities, for all people older than 12 years.

### Statistics

Raw data were sorted in Google Sheets and Microsoft Excel and analyzed by S.C.D.T. using IBM SPSS-Statistics version 27. The statistical significance level was set at *p* < 0.05.

All data were categorized into nominal variables except for the metric variables age, BMI, number of siblings, and fever duration. Age was categorized as follows: 0-6, 6-12, 12-18, and 18-24 months. For statistical analysis, detected pathogens were inventoried as a new variable “pathogen category”: rhino-/enterovirus, coronaviruses (coronaviruses 229E, HKU1, OC43, NL63), others (all single positive results without rhino-/enterovirus and coronaviruses), multiple infections (co-infections with more than one pathogen) or negative.

Descriptive statistics [median, interquartile range (IQR) Q1-Q3, mean, 95% confidence interval (CI)] were used to characterize the study population and recapitulate the number of weekly swabs. Shapiro-Wilk-Test was used to test for normal distribution. Weekly testing rates numbers in and out of lockdowns were compared using the unpaired Student's *t*-test. Analysis of qualitative values included absolute numbers and relative frequencies in percentage (%). Nominally scaled variables were tested using the Chi-Square test (*X*^2^). The association of two categorical variables was analyzed with the Cramér's V correlation coefficient, which was interpreted according to the Rea and Parker classification (*r* = 0.10-0.20 weak, *r* = 0.20-0.40 moderate, and *r* = 0.40-0.60 relatively strong association) (15). Multiple testing correction was done using the Bonferroni method, and adjusted *p*-values were calculated.

We performed multinomial logistic regression analysis to test the influence of diverse predictors (independent variables: age, sex, lockdown, siblings) on the occurrence of a specific pathogen—the nominal variable “pathogen category” being the dependent variable and the negative sample being the reference variable. Wald test was used to test for the significance of individual coefficients (16). Odds ratio (OR, 95% confidence interval and *p*-values were calculated.

## Results

### General Clinical and Socio-Demographic Characteristics of the Study Population

During the November 2020-April 2021 winter season, 390 children (0-24 months, 59% male, 41% female) with an acute respiratory infection or fever of unknown origin were included in this study. A total of 448 nasal swabs were collected, once in 340 children, twice in 43, thrice in 6 children, and four times in one child. The median age was 12 months (Q1-Q3, 6-18 months). Table 1 summarizes socio-demographic and general clinical characteristics.

**Table 1:** Characterization of study population.

**Age**	**Months**	**12 (6-18)**
BMI	Kg/m^2^	16.67 (15.8-18.3)
Sex	Male	230 (59)
	Female	160 (41)
Highest education mother	Compulsory education	58 (15)
	Apprenticeship	116 (30)
	High school diploma	70 (18)
	Academic degree	144 (37)
	Missing data	2 (<1)
Highest education father	Compulsory education	51 (13)
	Apprenticeship	130 (33)
	High school diploma	83 (21)
	Academic degree	121 (31)
	Missing data	5 (1)
Parents‘ marital status	In relation	343 (88)
	Separated	46 (12)
	Missing data	1 (<1)
Housing	Community housing	101 (25)
	Apartment	223 (57)
	House	64 (16)
	Missing data	2 (<1)
Chronic diseases	No	363 (93)
	Yes	26 (7)
	Missing information	1 (<1)

*Metric data are presented as median and (Q1-Q3), qualitative values as absolute numbers and relative frequencies in percentage (%). N = 390*.

The main clinical manifestations (in detail, refer to Table 2) that emerged from all 448 swabs were rhinitis (70%), followed by cough (64%). Symptomatic treatment included decongestant nasal sprays (45.5%), analgesics (57%), and antibiotics (10.9%). Inhalation therapy or systemic corticosteroids to treat laryngitis or bronchitis were rarely prescribed. Hospitalization was indicated only in one case (Table 2).

**Table 2:** Clinical characterization and medication.

**Clinical characteristics**	
Rhinitis	313 (70)
Nasal congestion	248 (55)
Fever	175 (39)
Cough	285 (64)
Pharyngitis	74 (16)
Fever duration in days	2 (1-3)
**Prescribed medication**	
Decongestant nose spray	204 (45)
Hypertonic saline nose spray	36 (8)
Decongestant and hypertonic saline nose spray	85 (19)
Analgesics	257 (57)
Antibiotics	49 (11)
Inhalation SABA	21 (5)
Inhaled corticosteroids	4 (<1)
Systemic corticosteroids	28 (6)
0.9% sodium chloride inhalation	4 (<1)
Hospitalization	1 (<1)

*Data are presented as absolute number and relative frequencies (%), qualitative data as median, (Q1-Q3). Multiple responses are possible as children experienced several symptoms and were prescribed more than one medication. N = 448 acute infections*.

### Non-SARS-CoV-2 Infections During the Pandemic

The Multiplex PCR was negative in 166 out of the 448 samples (37.1%), co-infections emerged in 21 samples (4.68%). The typical seasonal RSV and metapneumovirus pathogens were not detected in any of the study subjects during the whole recruitment period. Influenza A (H3) and influenza B were found in only one patient who had been vaccinated with a live-attenuated nasal flu vaccine (Fluenz tetra^®^) 1 week prior to the study recruitment.

**Figure 1:**
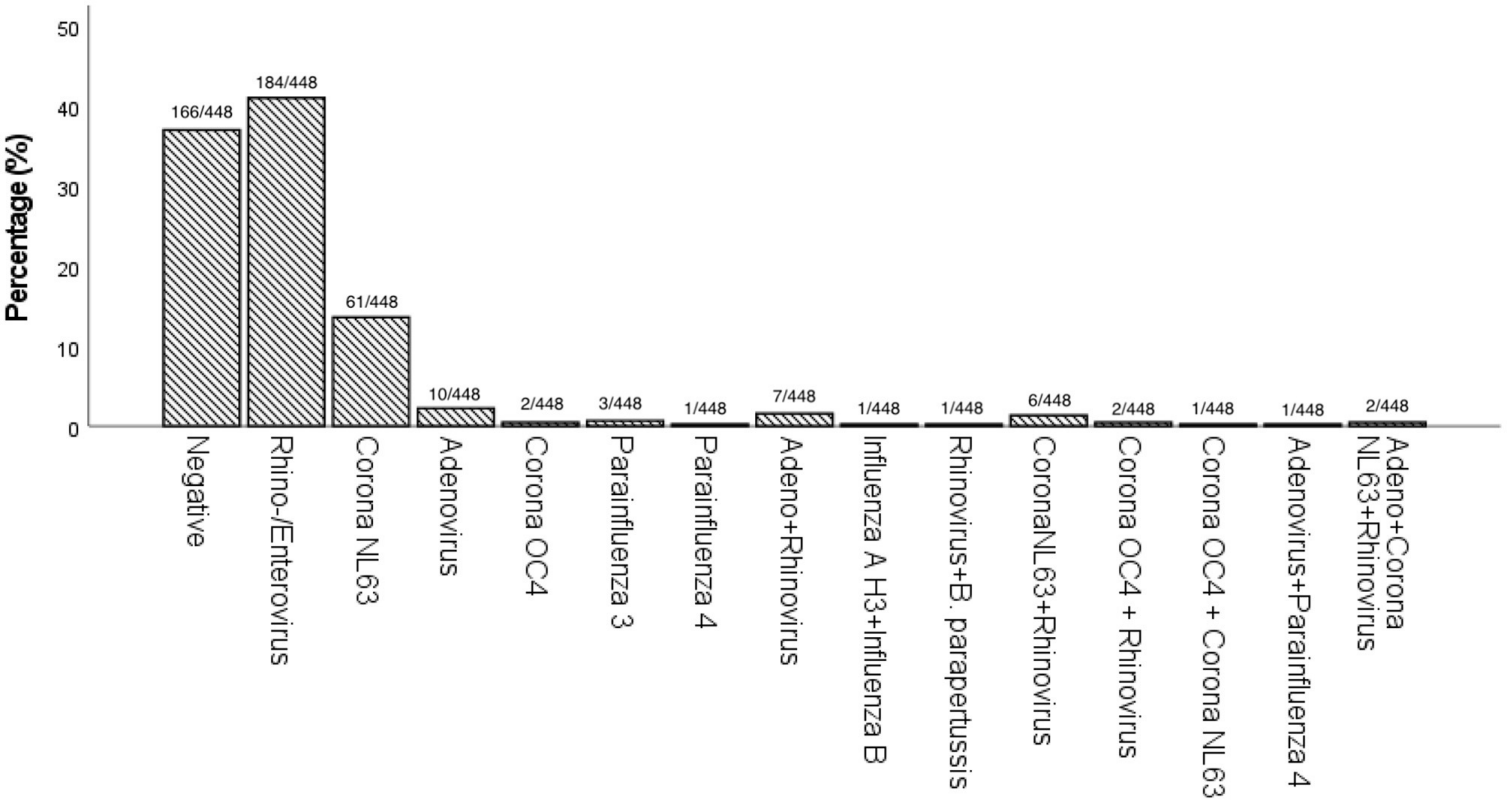
Prevalence (%) of pathogens detected in single and co-infections by Multiplex PCR.

The most prevalent pathogen was rhinovirus/enterovirus (*n* = 184, 41.1%), followed by coronavirus NL63 (*n* = 61, 13.6%), adenovirus (*n* = 10, 2.2%), parainfluenza virus 3 (*n* = 3, 0.7%), and coronavirus OC4 (*n* = 2, 0.4%) (Figure 1). One patient tested positive for Bordetella parapertussis, and was immediately treated with antibiotics as symptoms persisted.

As expected, rhino-/enterovirus infections were associated with nasal symptoms and cough, while coronaviruses infections predominantly led to cough and, to a lesser extent, nasal symptoms. In children with fever we rarely detected a respiratory pathogen (Table 3).

**Table 3:** Distribution of pathogens within the symptom category.

	**Rhinitis**		**Nasal**		**Fever**		**Cough**		**Pharyngitis**	
			**Congestion**							
	***n***	**%**	***n***	**%**	***n***	**%**	***n***	**%**	***n***	**%**
Negative	73	23%	73	29%	89	51%	72	25%	36	49%
Rhino-/enterovirus	168	54%	125	50%	42	24%	135	47%	24	32%
Coronaviruses	49	16%	32	13%	24	14%	54	19%	7	9%
Others	10	3%	4	2%	11	6%	6	2%	2	3%
Multiple infections	13	4%	14	6%	9	5%	18	6%	5	7%
Total number	313	100%	248	100%	175	100%	285	100%	74	100%
Cramér's V	0.462		0.242		0.315		0.358		0.125	
*P*-value	0.000		0.000		0.000		0.000		0.135	
*P*-adjust	<0.001		<0.001		<0.001		<0.001		0.675	

*Absolute number and Percentage (%) of symptom group. Multiple responses were possible. P-adj.: P corrected for multiple testing using Bonferroni method*.

The Chi-Square analysis determined a significant association between the pathogen groups and the observed symptoms for rhinitis (Cramér's V 0.462, *p*-adj. < 0.001), nasal congestion (Cramér's V 0.242, *p*-adj. < 0.001), fever (Cramér's V 0.315, *p*-adj. < 0.001), and cough (Cramér's V 0.358, *p*-adj. < 0.001).

### Longitudinal Analysis of Respiratory Pathogens Over the Winter Season

Our longitudinal, prospective study design allowed the delineation of the weekly prevalence of respiratory pathogens in symptomatic children during a pandemic and the trends' correlation with the Covid-19 regulations.

**Figure 2:**
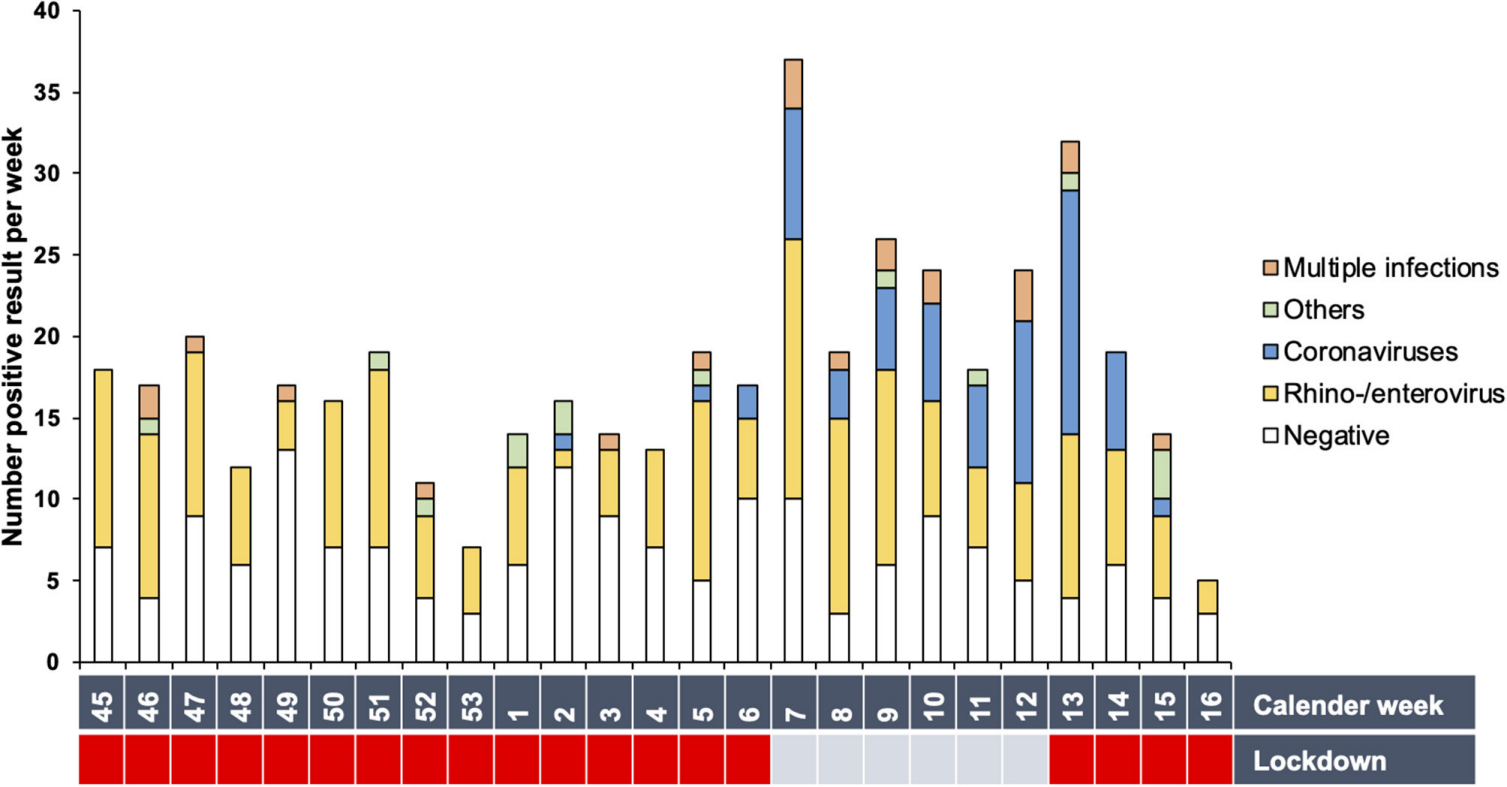
Prevalence of detected pathogens per calendar week. Total number of positive results per week are shown. White bars represent the number of negative results. The Covid-19 regulations are depicted as lockdown (red) or lockdown-free period (blue).

Rhino-/enterovirus and adenovirus were detected throughout the winter months despite the pandemic regulations. Coronavirus NL63 peaked in the first trimester of 2021 (Figure 2 and Supplementary Figure 1), especially during the weeks without a lockdown. Other pathogens were found only sporadically. The weekly number of nasal swabs, an indicator of the infection rate in the pediatric cohort, plummeted in the calendar weeks 45 to 4 and increased during the first trimester of 2021. Lockdown intervals registered considerably fewer swabs per week (average weekly number of swabs during lockdowns: 15.79 swabs/week, 95%CI (13.08,18.50) compared to weeks without a lockdown [24.66 swabs/week, 95%CI (17.53,31.80)], [*t*_(23)_ = 3.213, *p* = 0.004, *d* = 1.5].

### Predictors of Pathogen Category

We explored the potential association between specific predictors such as age group, siblings, and lockdown and the prevalence of a pathogen category using multinominal logistic regression analysis. There was only a significant association of the lockdown with coronaviruses (OR 4.371, 95% CI 2.34-8.14, p-adj. < 0.001), indicating a four times higher prevalence during lockdown-free periods. Lockdowns did not significantly influence any other detected pathogens (Supplementary Table 1).

## Discussion

This prospective, longitudinal cohort study analyzed the epidemiological profile of winter respiratory pathogens in infants and toddlers during Covid-19 pandemic lockdowns. Children (0-24 months) visiting Vienna's largest pediatric primary health care center due to symptoms of acute respiratory tract infection or with unexplained fever were mainly positive for rhino-/enterovirus, adenovirus, and coronaviruses; however, they were spared from influenza, RSV, and metapneumovirus throughout the winter season. The only positive influenza sample was caused by viral shedding in the recipient of a nasal flu vaccination 1 week prior to testing. This finding is consistent with publications indicating that PCR assays can detect viral shedding weeks after vaccination (17). The lockdown free periods showed a significant association with a higher risk for coronaviruses.

Our study cohort consisted of otherwise healthy term infants and toddlers. We explicitly excluded children with severe chronic or congenital disorders as this category is more likely to develop severe respiratory infections, e.g., due to RSV (18). These patients are regularly admitted to the hospital and might have a different risk profile regarding the exposure to infectious diseases than healthy children. We have chosen the 0-24 months age group because typically, Austrian children start kindergarten at around 3 years (19). As kindergartners do not wear masks or abide by social distancing, other hygiene rules are applied. This circumstance might alter the risk of exposure to infections and would have biased our research on the viral activity in children during a lockdown. Therefore, we expected our study cohort to be only indirectly affected by non-pharmaceutical Covid-19 measures.

In addition, we chose this age group specifically as respiratory infections caused by RSV, adenovirus, rhino-/enterovirus, and parainfluenza virus are significantly more common in the first years of life (20). Unfortunately, we are unable to compare our results with those from pre-Covid-19 research, as there are no similar studies conducted on the same age group in an Austrian pediatric primary health care setting before. However, based on international data and national surveillance systems that contain adult and pediatric results, it is clear that rhinovirus, influenza, RSV, parainfluenza, metapneumovirus, and adenovirus accounted internationally for up to 80% of acute respiratory tract infections (2, 21, 22) and were the culprit of respiratory infections before the pandemic also in Austria (10).

Several retrospectively designed studies investigated the impact of Covid-19 regulations on the occurrence of non-SARS-CoV-2 respiratory pathogens using surveillance systems data (10, 23, 24). An Austrian sentinel surveillance data analysis in spring 2020, when the country adopted the first Covid-19 measures, reported a significant decrease in influenza, RSV, metapneumovirus, and rhinovirus 1 week after the start of a lockdown (10). However, those data do not reflect the children's specific situation and do not provide any information about the patients' individual symptoms. What emerged from our study was, in fact, the occurrence of rhino-/enterovirus infections in children aged 0-24 months throughout the winter independent of the Covid-19 measures. An Australian surveillance study described a significant decline in RSV and influenza coinciding with the Covid-19 pandemic, while rhinovirus infections were above average. The authors hypothesized that the Covid-19 screening of asymptomatic patients and the absence of competition from influenza and RSV might have led to the higher numbers of rhinovirus detections (23). Other studies reported analogous high rhinovirus positivity rates during the Covid-19 pandemic and a drastic decline of influenza virus, human metapneumovirus, parainfluenza viruses, RSV, and adenovirus in children (25, 26).

Besides the absence of RSV, influenza, and metapneumovirus, we observed an association between lockdown and coronaviruses infection rates. The coronavirus NL63 peak in our study cohort was concomitant with the lockdown-free period in the first trimester of 2021. Under normal circumstances, the seasonality of human coronavirus peaks in the winter and spring months (27). A Finnish register-based study did not find any direct impact of reopening of schools and day care centers on the incidences of respiratory pathogens; however, researchers did not investigate for coronaviruses infections (28).

We tried to contextualize our results in the frame of the national Covid-19 mitigation measures because they substantially affected the social life of the whole family. However, infants and toddlers were only indirectly affected by these non-pharmaceutical measures, but might suffer the consequences of social isolation. In addition, their older siblings, who are often the source for respiratory infections for young children, might have brought less infections to their homes (29). Our data might corroborate this hypothesis as we did not observe a significant association between having siblings and the onset of specific respiratory or multiple infections. It might be speculated that children *per se* were less often ill during the pandemic or stayed at home more often with mild disease. Considering our results in relation to the Covid-19 lockdowns, the limited number of nasal swab samples collected during lockdown periods might point to the effects Covid-19 regulations could have on the respiratory infection rates in the pediatric cohort. Other studies confirm this hypothesis as acute otitis media, gastroenteritis, common cold, and bronchiolitis have also decreased among other pediatric populations (30, 31). The Covid-19 mitigation measures changed not only the circulation patterns of respiratory pathogens but seemed to affect the hospitalization rate. Only one of our study subjects was referred to the hospital. Analogous records on hospitalizations for acute bronchiolitis come from international studies (32, 33). RSV is usually the pathogen with the highest severity of infection and the primary cause of hospitalization and PICU admissions in young children (34, 35). Lockdown and school closures significantly correlated with reduced hospitalizations and overall pediatric visits rates in other countries (33, 35–38).

Ultimately, the question remains, why is one pathogen more affected than the other? One hypothesis focuses on the effects Covid-19 measures have on the specific transmission routes. While RSV, influenza, and coronaviruses are mainly airborne, adenoviruses and rhino-/enteroviruses can also be transmitted by direct contact (20, 39). In addition to that, other factors, such as travel restrictions and a higher vaccination coverage for influenza, might have inhibited the transmission during the study period (40). However, these assumptions require further investigation.

### Limitations

Although our practice regularly received patients with respiratory infections throughout the pandemic winter season, many families avoided in-person visits either because of quarantine or fear of Covid-19, which is confirmed by significant lower numbers of patient visits in the recruitment months 2020 compared to previous years (21.000 vs. 27.000 (November 2018– April 2019)). Most of the families were willing to take part in this study, but we did not analyse the rate of refusal to participate in detail. It is therefore impossible to ascertain a significant effect on single pathogens. It is plausible that some of our patients had a SARS-CoV-2 infection or co-infection when the swab was collected (41).

During the study period, a separate system other than the primary health care centers was in charge of testing all patients with typical Covid-19 symptoms for SARS-CoV-2 in Austria. Therefore, the SARS-CoV-2 analysis would have led to a biased underrepresentation of this coronavirus in our study cohort. In addition, since the detection of SARS-CoV-2 was not the focus of this study and considering Covid-19 rarely occurred among young children (7, 38), who are often asymptomatic or mildly affected, we consider this bias a minor limitation in our study (42).

Another limiting factor could be the single-center design of our study. Since our health care facility is Austria's largest primary pediatric practice, and our conclusions support the national surveillance data (5, 6), it can be assumed that our data is fairly representative.

Based on our clinical results fever is most often found in children with negative swab samples implying that this parameter is not a precise inclusion criterion for children with airway infections. Other viral pathogens, such as HHV6, are quite common to cause fever in this age group but was not included in our panel analysis.

We chose nasal instead of nasopharyngeal swabs for a less invasive approach that yields satisfactory results, as other studies suggest (43–45). However, we cannot exclude that our sampling technique led to lower detection rates. Using this non-invasive technique in future winter seasons will provide more information on the typical distribution patterns of respiratory viruses in our study cohort, as the lack of comparability might constitute a limitation in the current study.

Further information on the socio-demographic background (e.g., attending childcare or other family members with respiratory tract infections) might glean valuable insight into influencing factors, and should be considered in future studies on this topic.

We are aware that our study has some limitations but we see these data as an important starting point for future research, which might give more insights in the upcoming years.

## Conclusion

Our prospective longitudinal study shows that during the winter season, Covid-19 measures suppressed the seasonal activity of influenza, RSV, and metapneumovirus, but not of rhino-/enteroviruses among 0-2-year-old children in a pediatric primary health care setting. The lockdown measures were negatively associated with coronaviruses infections. We support the conclusions from retrospective surveillance studies about the influence of Covid-19 mitigation measures on the occurrence of other respiratory pathogens in children. However, further research is required to clarify the exact influence a particular measure has on a specific pathogen. This effort could be a crucial advancement in public health responses to future viral outbreaks.

## Data Availability Statement

The raw data supporting the conclusions of this article will be made available by the authors, without undue reservation.

## Ethics Statement

The studies involving human participants were reviewed and approved by the Ethics Committee of the Medical University of Vienna (EK-Nr. 1864/2020). Written informed consent to participate in this study was provided by the participants' legal guardian/next of kin.

## Author Contributions

SD-T conceptualized and designed the study, supervised data collection, carried out data analysis, drafted the initial manuscript, and reviewed and revised the manuscript. PV conceptualized and designed the study, supervised data collection, and reviewed and revised the manuscript. JV, KL, AR, UK, and PP recruited patients, collected data, and critically reviewed and revised the manuscript. AM-R and BM were responsible for supervision and interpretation of PCR analyses, and critically reviewed and revised the final manuscript. All authors approved the final manuscript as submitted and agreed to be accountable for all aspects of the work.

## Funding

This work was supported by MSD's Merck investigator Studies Program [MISP 60230]. The funders had no role in study design, data collection and analysis, decision to publish, or manuscript preparation.

## Conflict of Interest

The authors declare that the research was conducted in the absence of any commercial or financial relationships that could be construed as a potential conflict of interest.

## Publisher's Note

All claims expressed in this article are solely those of the authors and do not necessarily represent those of their affiliated organizations, or those of the publisher, the editors and the reviewers. Any product that may be evaluated in this article, or claim that may be made by its manufacturer, is not guaranteed or endorsed by the publisher.

## Supplementary Material

Supplementary Figure 1 Detailed prevalence of detected pathogens per calendar week. Total number of all positive results per week are shown without pathogen grouping. White bars represent the number of negative results. The Covid-19 regulations are depicted as lockdown (red) or lockdown-free period (blue).
