# Supplementary figures and images for: Respiratory Infections in Children During a Covid-19 Pandemic Winter
Source: Front Pediatr. 2021 Oct 18;9:740785. doi: 10.3389/fped.2021.740785 (PMC8558488; doi:10.3389/fped.2021.740785)

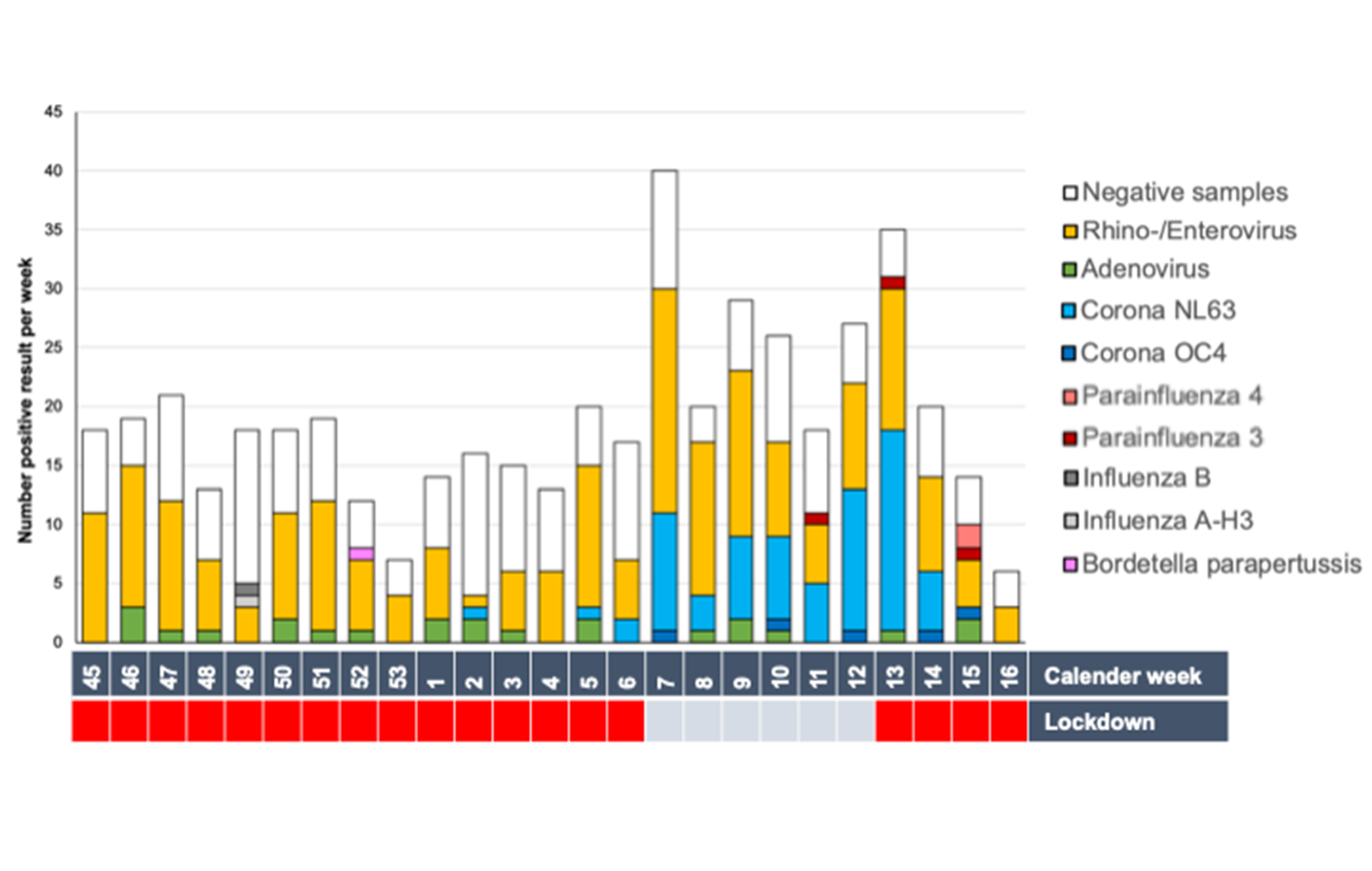

Supplement: Supplementary Figure 1 — Detailed prevalence of detected pathogens per calendar week. Total number of all positive results per week are shown without pathogen grouping. White bars represent the number of negative results. The Covid-19 regulations are depicted as lockdown (red) or lockdown-free period (blue). [file Image_1.TIFF]
