# Supplementary material for: Respiratory Infections in Children During a Covid-19 Pandemic Winter
Source: Front Pediatr. 2021 Oct 18;9:740785. doi: 10.3389/fped.2021.740785 (PMC8558488; doi:10.3389/fped.2021.740785)
Supplement: Supplementary file 2 [file Table_1.DOCX]

| **Supplementary Table 1. Association of lockdown and pathogen group** | | | | |
| --- | --- | --- | --- | --- |
|  | OR | 95% CI | p-value | p-adj. |
| Rhino-/enterovirus | 1.476 | (0.91-2.394) | 0.115 | 1 |
| Coronaviruses | 4.376 | (2.351-8.145) | <0.001 | **<0.001** |
| Others | 0.475 | (0.1-2.251) | 0.348 | 1 |
| Multiple Infections | 3.374 | (1.284-8.87) | 0.014 | 0.322 |
| Abbreviations: Odds ratio (OR), p-adj.: p-value after Bonferroni correction | | | | |
